# TST positivity in household contacts of tuberculosis patients: a case-contact study in Malawi

**DOI:** 10.1186/s12879-017-2348-2

**Published:** 2017-04-11

**Authors:** Jonas Hector, Suzanne T. Anderson, Gertrude Banda, Mercy Kamdolozi, Laura F. Jefferys, Doris Shani, Natalie J. Garton, Agnes Mwale, Annie Jobe, Geraint R. Davies, Derek J Sloan

**Affiliations:** 1 LSTM, Liverpool School of Tropical Medicine, Liverpool, UK; 2 Malawi Liverpool Wellcome Trust Clinical Research Programme, College of Medicine, University of Malawi, Blantyre, Malawi; 3 Section of Paediatrics and Imperial College–Wellcome Trust Centre for Global Health Research, Department of Medicine, Imperial College London, London, UK; 4 Department of Microbiology, College of Medicine, University of Malawi, Blantyre, Malawi; 5 Department of Infection, Immunity and Inflammation, University of Leicester, Leicester, UK; 6 Institute of Infection and Global Health, University of Liverpool, Liverpool, UK; 7 School of Medicine, University of St Andrews, Scotland, UK

**Keywords:** Tuberculosis, HIV status, Contact screening, Tuberculosis skin test, White blood cell count, Malawi, Lipid body

## Abstract

**Background:**

Screening household contacts of active tuberculosis (TB) patients is recommended for TB control. Due to resource constraints this rarely occurs in lower income countries. Demographic and clinical features of index cases may influence the likelihood of onwards TB transmission. It has also been proposed that accumulation of intracellular lipid bodies within *M. tuberculosis* cells may also enhance bacterial transmissibility. This study explored whether clinical and bacteriological observations recorded at baseline in TB cases in Malawi could help identify those with the highest risk of onwards transmission, to prioritise contact tracing.

**Methods:**

In this case-contact study, data on clinical presentation, sputum bacterial load and the percentage of lipid body positive acid-fast bacilli (%LB + AFB) on sputum smears were recorded in adults with sputum smear and culture positive pulmonary TB before initiation of therapy. The Tuberculin Skin Test (TST) was used to detect infection with *M. tuberculosis* amongst household contacts under the age of 15 years. TST positivity of the child contacts was related to characteristics of the index case.

**Results:**

Thirty four index cases brought 56 contacts (median: 1, range: 1–4 contacts each). 37 (66%) of contacts had a positive TST. Cavities or a high percentage of lung affected on index patient CXRs were associated with TST positivity. Multivariate analysis of non-radiological factors showed that male sex, HIV-negative status and raised peripheral blood white blood count (WBC) in index patients were also independent risk factors of TST positivity. Lower %LB + AFB counts were associated with TST positivity on univariate analysis only.

**Conclusion:**

TST positivity is common amongst household contacts of sputum smear positive adult TB patients in Malawi. Contact tracing in this high risk population could be guided by prioritising index cases with CXR cavities and extensive radiological disease or, in the absence of CXRs, those who are HIV-negative with a raised WBC.

## Background

Tuberculosis (TB) is a contagious, airborne disease that is linked to poverty. 1.5 million people died of TB in 2014 and the majority of deaths occured in resource-poor settings [1]. In Malawi where this study was conducted, the incidence of TB was 227/100,000 persons in 2014 [2]. Even though TB incidence is declining by 1.5% per year [1] transmission remains high, with children of known sputum smear positive (SSP) index cases at particular risk [3].

A cornerstone of a successful TB control program is tracing and screening of contacts of SSP patients in order to prevent new infections [4, 5]. Although active case finding in household contacts has proved to be effective in Malawi [6], the city of Blantyre alone notifies one thousand new SSP TB patients per year (unpublished Malawi TB Control Programme data). Under routine conditions, resource constraints prevent effective tracing for all of these [7]. Implementation of a structured approach to prioritise contact screening would be helpful, especially in settings where case detection rates remain low [8, 9].

Focussed contact tracing amongst patients with the highest probability of transmitting infection may optimise deployment of resources for maximal TB control [9]. It has previously been reported that HIV-negative patients with extensive radiological, particularly cavitary, disease and higher baseline bacterial burden in sputum are most likely to transmit infection [10–13]. Recently, bacterial phenotype has been proposed as a determinant of TB transmission [14]. Mycobacterium tuberculosis (Mtb) cells in a metabolically quiescent, non-replicating state may accumulate intracytoplasmic triacylglycerol lipid bodies (LB) which are identifiable by a modified sputum smear staining technique during fluorescence microscopy [14–16]. LB positive cells are believed to be robust when exposed to a variety of stressors, and some data suggest that this enhances their transmissibility [14]. However, clinical studies relating the proportion of LB-positive acid-fast bacilli (%LB + AFB) in sputum smears to infectiousness have never been undertaken.

This study aims to explore the extent to which clinical, radiological and microscopy-based bacteriological factors could help identify highly SSP index cases with the highest risk of onwards transmission, so that contact tracing efforts can be targeted in low resource settings. Identifying high-risk contacts could improve TB case detection and prevention.

## Methods

### Study design and population

This was a case – contact survey connected to two bigger studies. One investigated childhood TB and host RNA expression [17], the other investigated bacillary elimination rates and detection of bacillary LBs in sputum of pulmonary TB patients [18]. In this study we investigated Mtb infection in children <15 years old, who were household contacts of SSP TB patients. Mtb infection in contacts was detected using the Mantoux tuberculin skin test (TST). Features of the index case were analysed in relation to TST positivity results. The study was based in the urban setting of Queen Elizabeth Central Hospital (QECH) in Blantyre, Malawi during the period of March to June 2011.

### Index cases

Index cases were recruited from the TB service at QECH. Every patient with new SSP TB was considered for participation. We included adult patients aged 16–65 years who lived with children <15 years old and were willing to provide written, informed consent. We excluded index patients that had previous TB treatment in the last five years and those who lived with children who had previously been treated for TB or were known to already have a positive TST. The HIV status of each index case was documented and in cases of unknown status testing and counselling was done. A full blood count at baseline was performed according to local procedure.

Sputum smear status was assessed by Ziehl Neelsen (ZN) microscopy and graded according to standard methods [19]. For liquid culture, 1 ml of each specimen was decontaminated with N-acetly-L-cysteine/sodium hydroxide (NaOH) 3% and inoculated into Mycobacterial Growth Indicator Tubes (MGIT, Becton Dickinson). ZN microscopy and MGIT TBc Identification Test kits (Becton Dickinson) were used to confirm that positive cultures represented pure growth of Mtb and Days-to-positivity (DTP) were used to inversely represent bacterial growth.

Our previously published fluorescence microscopy method was used to quantify %LB + AFB counts for baseline sputum smears [18]. Ten microliter smears of lipase-dithiothreitol treated sputum smears were heat-fixed onto slides, flooded with auramine O for one minute, decolourised with 0.5% acid-alcohol for two minutes, reflooded with LipidTOX red neutral (LTR; Invitrogen, 1:200 dilution of stock solution in phosphate buffered saline) for 20 min and counterstained with 0.1% potassium permanganate for 45 s. Slides were washed in distilled water after each step and read at ×1000 magnification using an epifluorescence microscope with digital camera attachment. Smears were systematically scanned through a fluorescein isothiocyante filter and all fields containing auramine-stained, yellow-green, acid-fast-bacilli (AFB) were photographed. Identical fields were re-photographed through a tetramethylrhodamine filter to capture LTR-stained red LBs. AFB with ≥1 LB on paired images were classified as LB positive. %LB + AFB counts were allocated to each slide as follows:

$$ \%\mathrm{LB}+\mathrm{AFB}=100\left(\frac{\mathrm{Total}\ \mathrm{LB}\ \mathrm{positive}\ \mathrm{AFB}\ \mathrm{on}\ \mathrm{all}\ \mathrm{images}}{\mathrm{Total}\ \mathrm{AFB}\ \mathrm{on}\ \mathrm{all}\ \mathrm{ages}}\right) $$

Duplicate smears were made from each specimen and results expressed as mean %LB + AFB counts. Microscopic images were assessed by two independent readers.

A conventional chest x-ray (CXR) of each index case was obtained at base line. In accordance with a previously published method, the amount of lungfield affected by TB was graded by visual estimation of the extent of opacification, cavitation or other pathology and expressed as a percentage of visible lung. This assessment was based on the proportion of observed lung-field which looked abnormal and the density of abnormal opacification in abnormal areas. The presence or absence of cavities <4 cm or ≥4 cm diameter was recorded [20]. Each CXR film was read separately by a radiologist and an infectious disease specialist. If rated differently consensus was reached by discussion.

### Childhood contacts

The household contacts of each index case were referred to the paediatric TB clinic at QECH. All children were screened for TB using a TST and for HIV seropositivity. A TST induration of more than 10 mm or 5 mm (in HIV uninfected or infected child respectively) after 48–72 h was classified as positive [21].

Age, sex, HIV status, relationship to the index case and sleeping site in relation to the index case were also recorded for each contact.

### Statistical methods

Statistical analyses were performed in R version 3.2.1 [22]. Logistic regression was undertaken to assess the effect of radiological and non-radiological characteristics of index cases on TST positivity of contacts, with incorporation of random effects to account for non-independence of some observations (clustering od contacts within households of the same index patient). Co-variates for the multivariate analysis of non-radiological factors done using all variables with *p* < 0.1 on univariate testing, and variables for which there was a high a priori suspicion of effect (i.e. DPT on MGIT culture). Results were expressed as Odds Ratios (OR) with 95% confidence intervals (CI). Logistic and linear regression were used to investigate demographic and clinical characteristics contributing to cavitary disease or higher amount of lungfield affected on CXR. The Kruskall-Wallis test was used to assess the relationship between WBC and CXR cavities.

## Results

Thirty-four SSP TB patients brought their child contacts for screening and gave informed consent to participate in the study. The 34 index cases produced 56 child contacts, with a median of one (range: 1–4) contacts per index case.

### Characteristics of the index cases

Baseline characteristics of the 34 index cases are shown in Table 1. Twenty (58.8%) patients were male with a median age of 31 years (interquartile range [IQR]: 25–34 years). Twenty-four (72.7%) were HIV-positive, with a median CD4 count of 173 cells/μl (IQR: 115–360 cells/μl). The median WBC count of index patients was 6.6 × 10^3^ cells/μl (IQR: 5.4–7.6 cells/μl).

**Table 1:** Baseline characteristics of index patients

Index case characteristics	*N* = 34
Male sex, *n* (%)	20 (58.8%)
Age in years, median (IQR)	31 (25–34)
Body mass index in kg/m^2^, median (IQR)	19.2 (17.3–20.4)
Duration of cough in weeks, median (IQR)	8 (4–12)
Smoker, *n* (%)	5 (14.7%)
Peripheral blood WBC count in cells/μl median (IQR)	6.6 (5.4–7.6)
HIV positive, n (%)	24 (72.7%)
CD4 count of HIV positive patients, median (IQR)	173 (115–360)
HIV positive patients on ART at baseline, *n* (%)	8 (38.1%)
Cavities on baseline CXR, n (%)	
No cavity	12 (36.4%)
Cavity <4 cm diameter	12 (36.4%)
Cavity ≥4 cm diameter	9 (27.3%)
Amount (%) of abnormal lungfield on CXR, median (IQR)	25 (18–35)
Index smear status, *n* (%)	
+	2 (5.9%)
++	2 (5.9%)
+++	30 (88.2%)
Days-to-positivity on sputum culture, median (IQR)	4 (3.5–6.5)
%LB + AFB count, median (IQR)	28.3 (17.1–48.0)

The median amount of lung affected on CXR was 25% (IQR: 18–35%). Twelve (36.4%) patients had cavities <4 cm and 9 (27.3%) had cavities ≥4 cm diameter on CXR.

All patients were SSP; 30 (88.2%) were graded smear “+++”, 2 (5.9%) were “++” and 2 (5.9%) were “+” [23]. All patients were positive on MGIT sputum culture, with a median DTP of 4 days (IQR: 3.5–6.5 days). The median %LB + AFB count was 28.3% (IQR: 17.1–48.0%).

### Characteristics of the contacts

Thirty-two (59.3%) of the 56 child contacts were male with a median age of five years (IQR: 3–7 years). Two (4.0%) were HIV infected. History of prior BCG vaccination was incompletely recorded, but all 28 children with this information available had been vaccinated. Thirty (65.2%) child contacts slept in the same room as the index patient. TST was performed on all of the child contacts and 37 (66.1%) had a positive TST indicating TB infection.

### Association of index case CXR appearance with TST positivity in contacts

Sputum smear status is a recognised driver of TB infectiousness. However, 88.2% of our index cases were smear “+++”, precluding discrimination of infection risk on the basis of sputum microscopy and facilitating evaluation of additional transmission factors in this high risk cohort.

Table 2 confirms that CXR appearance strongly predicted TST positivity. The OR for TB infection in contacts of patients with cavities <4 cm was 7.58 (95% CI: 1.63–34.90) and the OR for infection with cavities ≥4 cm was 12.25 (95% CI: 2.20–68.24). The OR for TB infection was 1.05 (95% CI: 1.00–1.11) for each percentage point rise in amount of lung-field affected.

**Table 2:** Assocation with index case CXR appearance with TST positivity in contacts

Index CXR feature	OR of positive TST in contacts (95% CI)
Cavities (ref = no cavity)	
Cavity <4 cm diameter	7.58 (1.63–34.90)
Cavity ≥4 cm	12.25 (2.20–68.24)
Amount (%) of abnormal lungfield	1.05 (1.00–1.11)

**Fig. 1:**
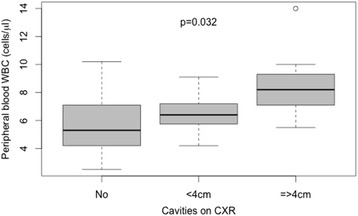
Relationship between peripheral blood WBC and cavitary disease on CXR assessed by Kruskall-Wallis test

Logistic and linear regression analyses were run to assess factors contributing to radiological presentation. On univariate assessment of this small sample, male sex was the only factor contributing to higher likelihood of cavitary disease (OR: 9.6 [95% CI: 1.85–49.88) and duration of cough prior to diagnosis was the only factor contributing to high percentage of lung field affected (Regression Co-efficient per week of cough: 0.96 [95% CI: 002–1.89]). Multivariate analyses were not performed due to the high level of co-linearity between the radiological features. There was a trend towards cavitary disease in patients with higher peripheral blood WBC, and patients with higher WBCs were likely to form bigger cavities (Fig. 1) suggesting that systemic inflammation might be a discriminatory driver of CXR appearance and TB transmission in SSP patients.

### Association of non-radiological features of index cases and TST positivity in contacts

Although CXR appearance can discriminate transmission risk between SSP patients, many TB diagnoses in Malawi are made at Primary Health Centres where radiology facilities are unavailable. Therefore logistic regression analysis were run to identify non-radiological features which may predict TB transmission risk and help prioritise SSP contact tracing in high burden settings without CXR.

Table 3 shows that on univariate analysis, male sex, higher peripheral blood WBC and lower %LB + AFB count in sputum all predicted higher likelihood of a positive TST in child contacts. A strong trend to positive TST was associated with HIV negativity. On multivariate analysis, male sex (OR: 7.62 [95% CI: 1.14–50.77]), a negative HIV test (OR:25 [95% CI: 1.26–100]) and higher WBC (OR: 1.73 [95% CI: 1.01–2.94]) remained statistically significant suggesting that these are independent risk factors for transmission.

**Table 3:** Relationship of non-radiological features of index cases to TST positivity in contacts

Index case characteristics	OR of positive TST in contact (95% CI)	
	Univariate analysis	Multivariate analysis^a^
Male sex	4.05 (1.26–13.04)	7.62 (1.14–50.77)
Age in years	0.96 (0.88–1.04)	-
Body mass index in kg/m^2^	1.06 (0.84–1.34)	-
Duration of cough in weeks	1.04 (0.93–1.16)	-
Smoker	0.75 (0.11–4.92)	-
Peripheral blood WBC	1.44 (1.03–2.01)	1.73 (1.01–2.94)
HIV negative	8.25 (0.97–69.87)	25 (1.26–100)
Days to positivity on sputum culture	0.95 (0.80–1.13)	0.96 (0.67–1.38)
%LB + AFB count	0.97 (0.95–1.00)	1.02 (0.97–1.06)

^a^Multivariate ORs including random effects to account for non-independence of some observations

## Discussion

In this case-contact investigation of adults with sputum culture confirmed TB in Blantyre, Malawi, TST positivity among the child contacts was common (66%). The observed rate of TST positivity was substantially higher than the rate previously described by Sinfeld et al., who described 45% TST positivity in child contacts in Malawi [24], and much higher than 9–12% baseline rate of TST positivity reported in Malawian schoolchildren [25]. However, as our adult study participants were strongly SSP, the finding of a higher rate of TST positivity amongst childhood contacts was not surprising. Improved contact tracing to identify infected child contacts of SSP adult TB cases is required, and tools to prioritise the highest risk individuals would be useful [26].

The observed association between cavitary disease or amount of lung-field affected on CXR of the index case and TST positivity of the child contact is consistent with findings from other studies. [13, 27, 28]. However, prior studies have not focussed on discriminatory analysis amongst strongly SSP patients and our data also illustrates that extent of radiological disease can usefully triage infectiousness amongst a subset of individuals who are all known to have multi-bacillary disease. As inflammation plays a role in the pathogenesis of TB [29] the trend towards greater cavitation in index patients with higher WBC in our study could indicate that systemic inflammation may play an important role in the pathophysiology of TB transmission.

As CXR is not routinely performed on new TB patients in Malawi, particularly at Primary Health Centres where TB registration increasingly occurs, it was important to also assess non-radiological predictors of infectiousness. Male sex, a negative HIV test and higher WBC were all independently associated with a positive TST in child contacts. Prior evidence on the role of HIV status in infectiousness is mixed [26, 30–33]. To our knowledge, this is the first time higher WBC has been linked to infectivity of index TB although previously, higher WBC has been associated with a delayed treatment response and poorer outcomes [34, 35]. These data support the observation that a strong systemic inflammatory response is implicated in transmission. They indicate that, even without radiology, demographic observations and simple blood tests may be used to triage transmission risk from SSP TB patients.

On univariate analysis, a lower %LB + AFB count was associated with higher likelihood of a positive TST in child contacts. This was contrary to the original hypothesis that physiologically robust, metabolically quiescent LB-positive cells may be well adapted for TB transmission. An alternative thesis, and possible explanation for our findings, is that metabolically quiescent bacteria, expressing fewer antigens, may be less likely to provoke the systemic inflammation and cavity formation needed for high-level infectiousness. As this pilot study was small, and the univariate effect was lost on multivariate analysis, clear conclusions cannot be drawn. There is scope however, for further investigation of the relationship between Mtb lipid metabolism and disease transmission.

There were a number of limitations to this study. As the sample size was small, these results should be viewed as preliminary analyses which require to be confirmed in larger studies. In a high burden setting, it is possible that some children may have been infected with TB from a source other than the index case identified here.

We note that the index cases brought a mean of 1.65 children (range 1–4) from their households to be screened. This number of children is lower than the number of children per household that can be calculated from the Malawi Demographic and Health Survey 2010. In the Report the mean household size is stated as 4.6 with 45.3% persons of the household being below the age of 15 years which equal a mean of 2 children under the age of 15 [36]. A selection bias could have been introduced by caregivers bringing children that they considered in need of medical attention whilst leaving seemingly well children in the home. This possibility requires further investigation as it has implications for further contact tracing studies in our setting.

Despite blinded double-reading, CXR and %LB + AFB assessments are subjective measurements with potential for variable interpretation. It was not possible to fully account for any confounding effect of infant BCG vaccination (although the data we have suggests that vaccine coverage was comprehensive and would not have biased results). TSTs were not augmented by Interferon gamma release assays due to resource constraints.

Nevertheless, our study highlights the need for tools to optimise TB contact tracing in high burden settings. It would be preferable to perform contact tracing on child contacts of all new SSP adult TB patients. However, when comprehensive coverage is not possible, better understanding of additional factors which contribute to disease transmission is important [37] Our finding that simple markers of systemic inflammation, such as more severe CXR abnormalities or a raised WBC, increases the likelihood of TB transmission could potentially help prioritise contact tracing efforts.

Overall, although these exploratory findings on a limited sample size must be interpreted with caution they do provide some insights into variability in TB transmission from the highest risk group of strongly sputum smear positive index cases with highest bacillary load. The data presented will be thought-provoking for those with an interest in contact-tracing and will inform plans for future research.

## Conclusion

The high TST positivity seen in child contacts of SSP adult TB patients highlights the importance of contact tracing. Data from our study suggests that cavities or extensive disease on CXR could help identify high-risk of onwards transmission. Furthermore if CXR is unavailable, male sex, HIV-negative status and high peripheral blood WBC might be associated with elevated TB infectiousness. Whilst fluorescence microscopy quantifies the proportion of LB + AFB in sputum, in this small study we could not establish a link to transmission.

## Abbreviations

AFB: Acid-fast-bacilli
BCG: Bacillus Calmette–Guérin
CI: Confidence Interval
CXR: Chest x-ray
DTP: Days to positivity
IGRA: Interferon gamma release essay
IQR: Inter quartile range
LP: lipid bodies
Mtb: Mycobacterium tuberculosis
OR: Odds ratio
QECH: Queen Elizabeth Central Hospital
SSP: Sputum smear positive
TB: Tuberculosis
TST: Tuberculosis skin test
WBC: White blood cell count
ZN: Ziehl Neelsen
%LB + AFB: percentage of lipid body positive acid-fast bacilli
